# The Effect of High Dose Cholecalciferol on Arterial Stiffness and Peripheral and Central Blood Pressure in Healthy Humans: A Randomized Controlled Trial

**DOI:** 10.1371/journal.pone.0160905

**Published:** 2016-08-10

**Authors:** Iain Bressendorff, Lisbet Brandi, Morten Schou, Birgitte Nygaard, Niels Erik Frandsen, Knud Rasmussen, Lars Ødum, Ove Vyff Østergaard, Ditte Hansen

**Affiliations:** 1 Department of Cardiology, Nephrology and Endocrinology, North Zealand Hospital, Hillerød, Denmark; 2 Department of Medicine, Roskilde Hospital, Roskilde, Denmark; 3 Department of Cardiology, Herlev and Gentofte Hospital, Herlev, Denmark; 4 Department of Clinical Biochemistry, Roskilde Hospital, Roskilde, Denmark; Indiana University Richard M Fairbanks School of Public Health, UNITED STATES

## Abstract

**Background:**

Low levels of serum 25-hydroxy vitamin D are associated with increased arterial stiffness and hypertension. Supplementation with vitamin D precursors has been proposed as a treatment option for these conditions. We examined the effect of oral cholecalciferol on arterial stiffness and blood pressure in healthy normotensive adults.

**Methods:**

40 healthy adults were randomised in this double-blinded study to either oral cholecalciferol 3000 IU/day or matching placebo and were followed for 16 weeks to examine any effects on pulse wave velocity (PWV), augmentation index (AIx), peripheral and central blood pressure and 24-hour ambulatory blood pressure.

**Results:**

22 subjects in the cholecalciferol arm and 18 subjects in the placebo arm completed the 16 weeks of follow-up. There was no difference in changes in PWV, AIx corrected for heart rate or central or peripheral blood pressure between the two groups. There was no correlation between serum 25-hydroxy vitamin D and any of these parameters.

**Conclusions:**

Oral cholecalciferol 3000 IU/day does not affect arterial stiffness or blood pressure after 16 weeks of treatment in healthy normotensive adults.

**Trial Registration:**

ClinicalTrials.gov NCT00952562

## Introduction

Serum 25-hydroxy vitamin D (25-OH vitamin D) levels are frequently low among healthy adults [1] and are associated with increased risk of cardiovascular events [2, 3] as well as the well-known effects on fractures and mineral homeostasis. There is some debate as to the adequate levels of 25-OH vitamin D for human health with some advocating levels above 75 nmol/L [4] and others levels above 50 nmol/L [5]. Previous studies have found an inverse relationship between serum 25-OH vitamin D and arterial stiffness in the form of increased pulse wave velocity (PWV) in healthy adults [6–9]. Studies have also found associations between low levels of 25-OH vitamin D and prevalence of hypertension [10], as well as risk of incident hypertension [11–13] although not all have found this association [14]. Because of these associations supplementation with vitamin D precursors (e.g. cholecalciferol or ergocalciferol) have been suggested as possible treatment options for treating or preventing these conditions in otherwise healthy normotensive adults. Indeed, 1,25-dihydroxy vitamin D (1,25-(OH)_2_ vitamin D) has been shown to have anti-inflammatory properties [15] and to inhibit the renin-angiotensin-aldosterone system (RAAS) [16], both of which are thought to prevent the development of arterial stiffness and hypertension. We hypothesized that treatment with high dose cholecalciferol would reduce arterial stiffness and blood pressure among healthy normotensive adults. In this study we present the secondary outcomes of a randomized double-blinded placebo-controlled trial of high dose cholecalciferol treatment in healthy normotensive adults with 25-OH vitamin D deficiency to see whether this treatment affects arterial stiffness or blood pressure.

## Methods

Subjects were recruited through posters and information meetings at Roskilde University Hospital. The study took place at the Department of Nephrology, Roskilde University Hospital, Denmark, in December 2009 to January 2010 and December 2010 to January 2011.

The study was performed in order to 1) describe changes in urinary calcium excretion and mineral metabolism (primary end-point, previously published along with trial protocol [17]) and 2) changes in arterial stiffness and blood pressure during cholecalciferol treatment in healthy adults (secondary end-points).

The details regarding the execution of this trial have previously been published [17]. Briefly, healthy adults (age >18 years) with serum 25-OH vitamin D ≤50 nmol/L not treated with medications that might influence mineral metabolism (vitamin D analogues or calcimimetics) or vascular parameters (antihypertensive or antidiabetic medication), and with no diseases affecting intestinal absorption or mineral metabolism (sarcoidosis, current cancer or cancer within 5 years of study enrolment, pancreatitis, malabsorption), were randomised in a 1:1 ratio to receive 3000 IU (75 μg) cholecalciferol orally once daily or placebo once daily for 16 weeks. At baseline and after 16 weeks of treatment PWV, pulse wave analysis, and 24-hour ambulatory blood pressure were measured as well as collection of fasting blood and urine samples.

The study was originally powered to detect a difference in urinary calcium excretion; however, according to previous studies a sample size of 10 subjects in each intervention group will have a power to detect a clinical relevant difference of 1 m/s in aortic PWV (α = 0.05) [18]. Therefore the sample size should be sufficient to detect a difference in this parameter.

The study is in compliance with the Helsinki Declaration of 1975, revised 1983, and approved by the Danish National Committee on Biomedical Research Ethics (SJ-135) and registered in ClinicalTrials.gov (NCT00952562). Written informed consent was obtained from all study participants.

### Laboratory analyses

Blood samples were collected for measurement of 25-OH vitamin D, 1,25-(OH)_2_ vitamin D and shipped cold and in total for analysis. Blood samples were taken at week 0 and week 16 when the subjects had fasted for at least 8 hours. Serum creatinine was measured concurrently by the same standardised analysis of the local laboratory.

Serum 25-OH vitamin D was determined by DiaSorin LIAISON 25 OHVitamin D assay, which is a direct competitive chemiluminescence immunoassay (CLIA) for quantitative determination of total 25-OH vitamin D in serum. Precision ranges (i.e. CVs) for the LIAISON assay were: within run (2.8–8.1%) and total precision (7.3–17.5%). Measuring range is 4.0 to 150 ng/mL.

Plasma 1,25-(OH)_2_ vitamin D was determined by a radioimmunoassay (Gamm-B1,25-dihdroxy Vitamin D, Immunodiagnostic Systems [IDS], Ltd.,Boldon, England). The CV was between 6.8% and 14.0% at plasma levels in the range of 16 to 220 pmol/L.

### Vascular parameters

#### Pulse wave velocity, pulse wave analysis and peripheral and central blood pressure

Measurement was performed by applanation tonometry. A pencil-shaped high-fidelity micromanometer registers the intra-arterial pulse-wave, when applied over a peripheral artery (a. radialis, a. carotis and a. femoralis).

A ten second recording of the arterial pressure in a. radialis was transformed to a central aortic waveform. This was done by the general transfer function in a validated software program, SphygmoCor^®^ (version 8.0, AtCor Medical, Sydney, Australia). The measurements were calibrated by the brachial blood pressure and the augmentation index (AIx) was then calculated from the central blood pressure curve. AIx is a measurement of the pulse wave amplification due to peripheral reflexion of the pulse wave. AIx is calculated as the difference between the first and second systolic peak as a percentage of the central pulse pressure (difference between central systolic and diastolic pressure). All AIx data in this trial were corrected for heart rate (AIx@HR).

Measurement of aortic pulse wave velocity was done by measurement of pressure waveforms in a. carotis and a. femoralis and a simultaneous electrocardiogram (ECG). The transit time was calculated as the time between the R-spike in the ECG and the arrival of the foot of the pulse wave (intersecting tangent) at the peripheral recording sites. The travel distance was measured by subtracting the carotid-suprasternal notch distance from the suprasternal notch-femoral distance [19].

All measurements were done in duplicate and the mean of the two measurements was recorded. The Sphygmocor software provides a quality control of the recorded pressure waveforms. If these control criteria were not met the measurement was discarded and replaced by a new measurement [20].

Measurements were performed after ten minutes rest in a calm environment and at a constant room temperature. The participants were not allowed to speak or sleep during the examination. The subjects were not allowed to eat, drink or smoke three hours before or consume any alcoholic beverage ten hours before the examination, according to the present guidelines [21].

#### 24-hour blood pressure

An ambulatory monitor, Spacelabs Medical, measured the 24-hour blood pressure every 15 minutes at daytime (6 AM to 10 PM) and every 30 minutes at night (10 PM to 6 AM) over a period of 24 hours.

### Statistical analyses

The biostatistical evaluation was carried out using SPSS version 22.0.0.0 (IBM Corporation, Armonk, NY, US). Continuous data were described as mean ± standard deviation (SD) and for differences the mean and 95% confidence interval (CI). All data was found to be of a Normal distribution. When comparing the two groups data was analysed by an unpaired t-test, and when analysing changes from baseline to week 16 in each group a paired t-test was used (matched pairs). Correlation between changes in 25-OH vitamin D and 1,25-(OH)_2_ vitamin D and changes in PWV, AIx@HR, 24-hour ambulatory systolic blood pressure (SBP), diastolic blood pressure (DBP) and pulse pressure (PP) were described by Pearson's correlation coefficient. All tests were two sided tests and Bonferroni correction was used to account for multiple testing, so the threshold for statistical significance was p < 0.0039 (0.05 divided by 13).

## Results

Fifty subjects were randomized and of these 40 subjects completed the study. All subjects were Caucasian. According to tablet count full compliance was achieved. Further details regarding drop-outs and side-effects have previously been published [17].

Baseline clinical and biochemical parameters (Table 1) as well as baseline vascular parameters (Table 2) were similar for the two groups, except for AIx@HR, which was slightly higher in the placebo group.

**Table 1:** Demographic Characteristics.

Table 1	Total (n = 40)	Cholecalciferol (n = 22)	Placebo (n = 18)
Male sex (%)	23 (57,5%)	11 (50%)	12 (66%)
Age (years ± SD)	42.6 ± 8.8	41.0 ± 9.05	44.5 ± 8.5
BMI (kg/m^2^ ± SD)	24.9 ± 4.0	24.7 ± 3.8	25.1 ± 4.3
eGFR (mL/min ± SD)	96.3 ± 12.7	97.7 ± 12.2	94.5 ± 13.4
25-OH vitamin D (nmol/L ± SD)	32 ± 10	31 ± 11	32 ± 10
1,25-(OH)_2_ vitamin D (pmol/L ± SD)	134.8 ± 32.1	129.2 ± 30.8	141.7 ± 33.2

BMI = Body Mass Index, eGFR = estimated Glomerular Filtration Rate, SD = Standard Deviation.

**Table 2:** Results of Cholecalciferol Treatment versus Placebo.

Table 2	Cholecalciferol group (n = 22)			Placebo group (n = 18)			Difference in changes betweencholecalciferol group and placebo group (95% CI)
	Baseline± SD	16 weeks± SD	Change from baseline to week 16 (SEM)	Baseline± SD	16 weeks± SD	Change from baseline to week 16 (SEM)	
25-(OH)-vitamin D (nmol/L)	31 ± 11	88 ± 20	57 (4)*	32 ± 10	37 ± 12	5 (2)	51 (40 to 62)*
1,25-(OH)_2_-vitamin D (pmol/L)	129 ± 31	165 ± 39	36 (8)*	142 ± 33	131 ± 22	-11 (7)	34 (13 to 55)*
PWV (m/s)	6.4 ± 1.4	6.4 ± 1.2	-0.0 (0.2)	6.7 ± 0.9	6.6 ± 0.6	-0.1 (0.1)	-0.2 (-0.9 to 0.4)
AIx@HR (%)	6.9 ± 3.6	6.9 ± 11.0	-0.0 (1.8)	10.6 ± 8.3	11.9 ± 10.4	1.3 (1.2)	-5.0 (-11.9 to 1.9)
aSBP (mmHg)	117.1 ± 11.1	117.3 ± 10.9	0.2 (0.8)	117.5 ± 8.3	115.8 ± 8.9	-1.7 (1.2)	1.5 (-4.9 to 8.0)
aDBP (mmHg)	72.9 ± 7.9	74.2 ± 7.9	1.3 (0.5)	73.4 ± 6.0	72.5 ± 5.6	-0.9 (0.8)	1.7 (-2.8 to 6.2)
aPP (mmHg)	44.2 ± 5.1	43.1 ± 5.3	-1.1 (0.5)	44.1 ± 5.3	43.4 ± 6.7	-0.6 (0.7)	-0.3 (-4.2 to 3.6)
pSBP (mmHg)	117.7 ± 11.1	115.3 ± 9.2	-2.5 (1.5)	118.0 ± 10.0	113.5 ± 10.5	-5.2 (1.4)*	1.8 (-4.6 to 8.2)
pDBP (mmHg)	72.1 ± 6.7	71.1 ± 8.3	-1.0 (1.6)	73.0 ± 5.8	71.4 ± 8.2	-1.8 (1.9)	-0.2 (-5.6 to 5.2)
pPP (mmHg)	45.6 ± 7.7	44.7 ± 8.4	-1.0 (1.6)	45.0 ± 10.4	41.5 ± 8.8	-3.9 (1.4)	3.2 (-2.5 to 8.8)
cSBP (mmHg)	105.1 ± 12.3	103.1 ± 10.8	-2.0 (1.7)	104.5 ± 16.9	103.5 ± 11.1	-1.0 (3.3)	-0.4 (-7.5 to 6.6)
cDBP (mmHg)	72.7 ± 6.8	71.9 ± 8.4	-0.8 (1.6)	71.9 ± 11.1	71.7 ± 8.0	-0.3 (2.7)	-0.3 (-5.0 to 5.6)
cPP (mmHg)	26.6 ± 4.7	26.2 ± 5.4	-0.3 (1.0)	26.6 ± 5.8	25.0 ± 5.1	-1.6 (0.8)	1.2 (-2.1 to 4.6)

* p < 0.0039

aDBP = 24-hour Ambulatory Diastolic Blood Pressure, aPP = 24-hour Ambulatory Pulse Pressure, aSBP = 24-hour Ambulatory Systolic Blood Pressure, AIx@HR = Augmentation Index adjusted for Heart Rate, cDBP = Central Diastolic Blood Pressure, CI = Confidence Interval, cPP = Central Pulse Pressure, cSBP = Central Systolic Blood Pressure, pDBP = Peripheral Diastolic Blood Pressure, pPP = Peripheral Pulse Pressure, pSBP = Peripheral Systolic Blood Pressure, PWV = Pulse Wave Velocity, SD = Standard Deviation, SEM = Standard Error of the Mean.

**Fig 1:**
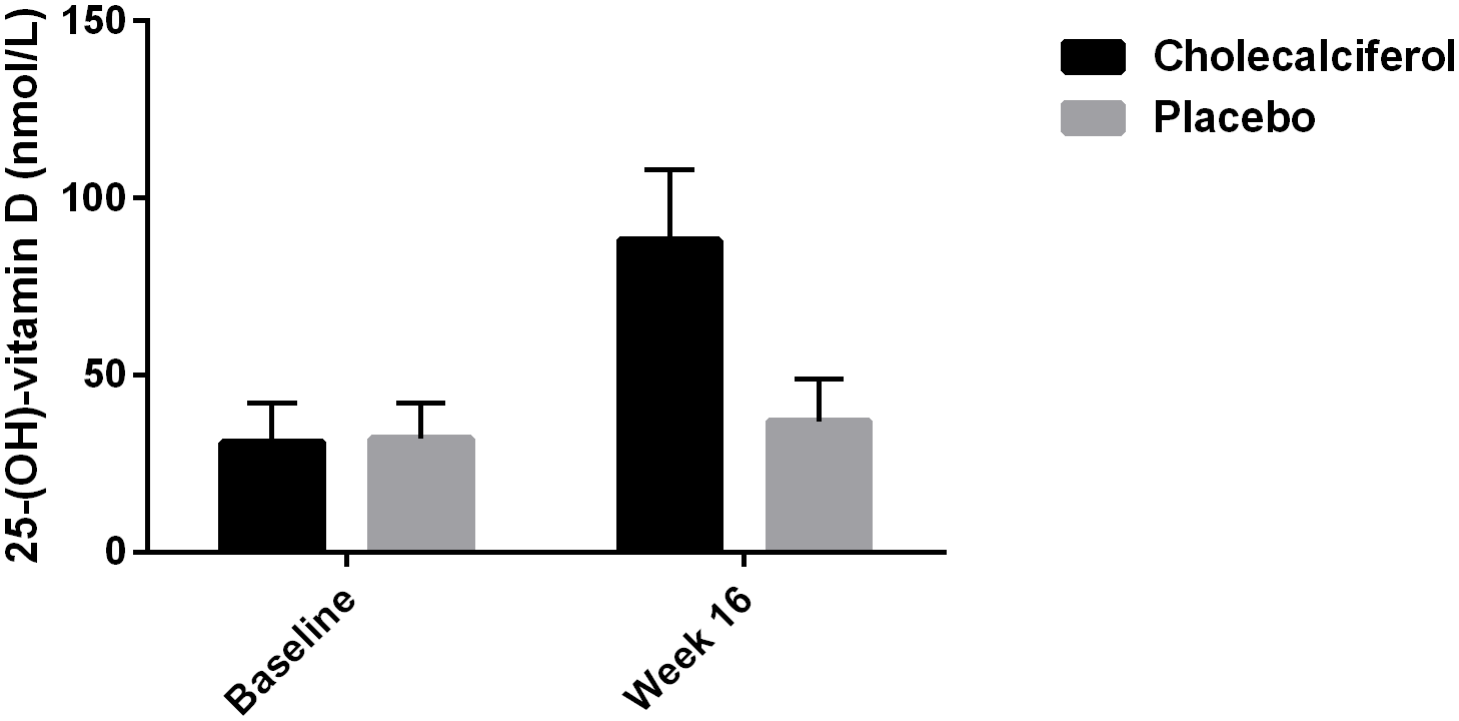
Treatment Effect on 25-OH Vitamin D Levels.

**Fig 2:**
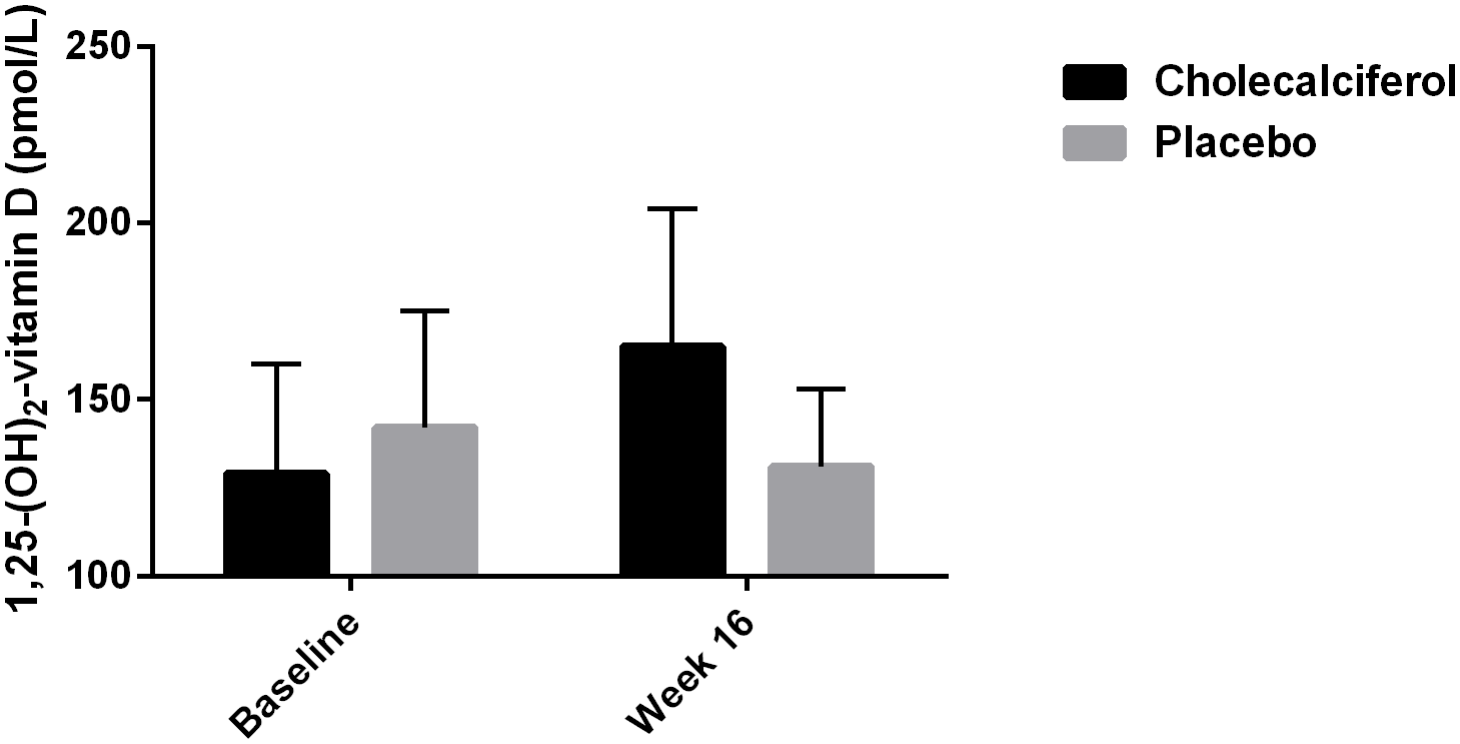
Treatment Effect on 1,25-(OH)_2_ Vitamin D Levels.

Changes in biochemical and vascular parameters are shown in Table 2. After 16 weeks of treatment there was a statistically significant difference in 25-OH vitamin D of 51 nmol/L (95% CI: 40 to 62, p < 0.0001) (Fig 1), as well as a statistically significant difference in 1,25-(OH)_2_ vitamin D of 34 pmol/L (95% CI: 13 to 55, p < 0.002) between the two groups (Fig 2). In paired samples analyses 25-OH vitamin D and 1,25-(OH)_2_ vitamin D increased significantly in the cholecalciferol group by 57 nmol/L (95% CI: 49 to 66, p < 0.0001) and 36 pmol/L (95% CI: 18 to 53, p < 0.0001), respectively. Further details of the treatment effects on markers of mineral metabolism have previously been published [17].

**Fig 3:**
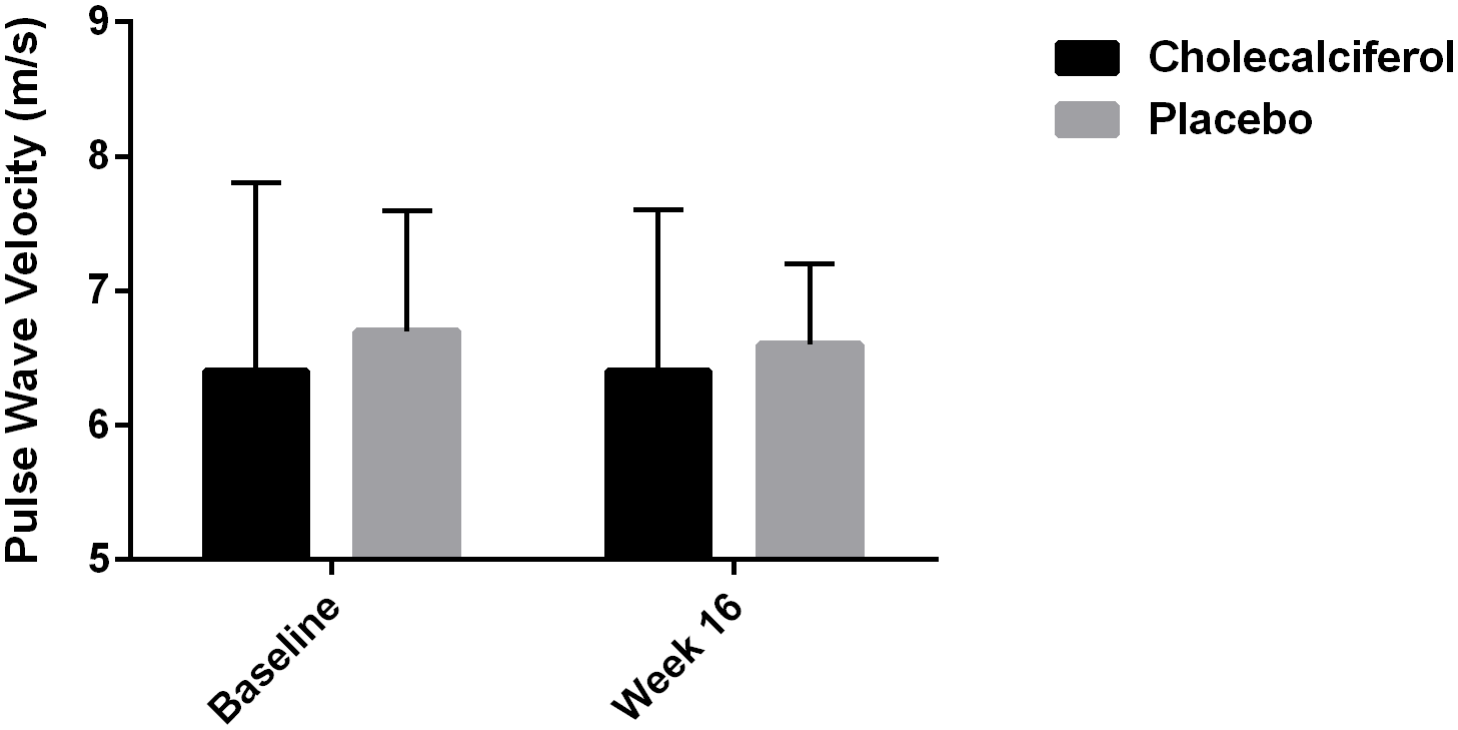
Treatment Effect on Pulse Wave Velocity.

**Fig 4:**
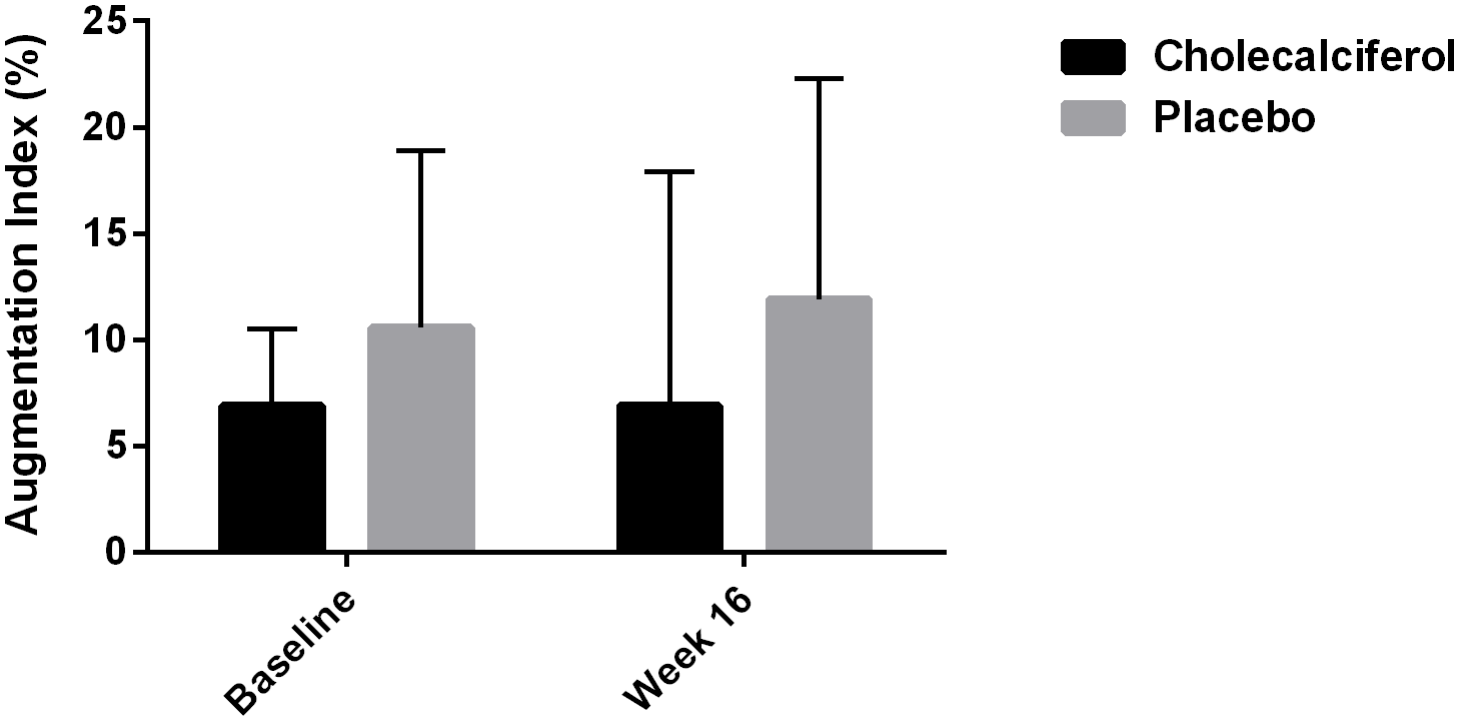
Treatment Effect on Augmentation Index.

**Fig 5:**
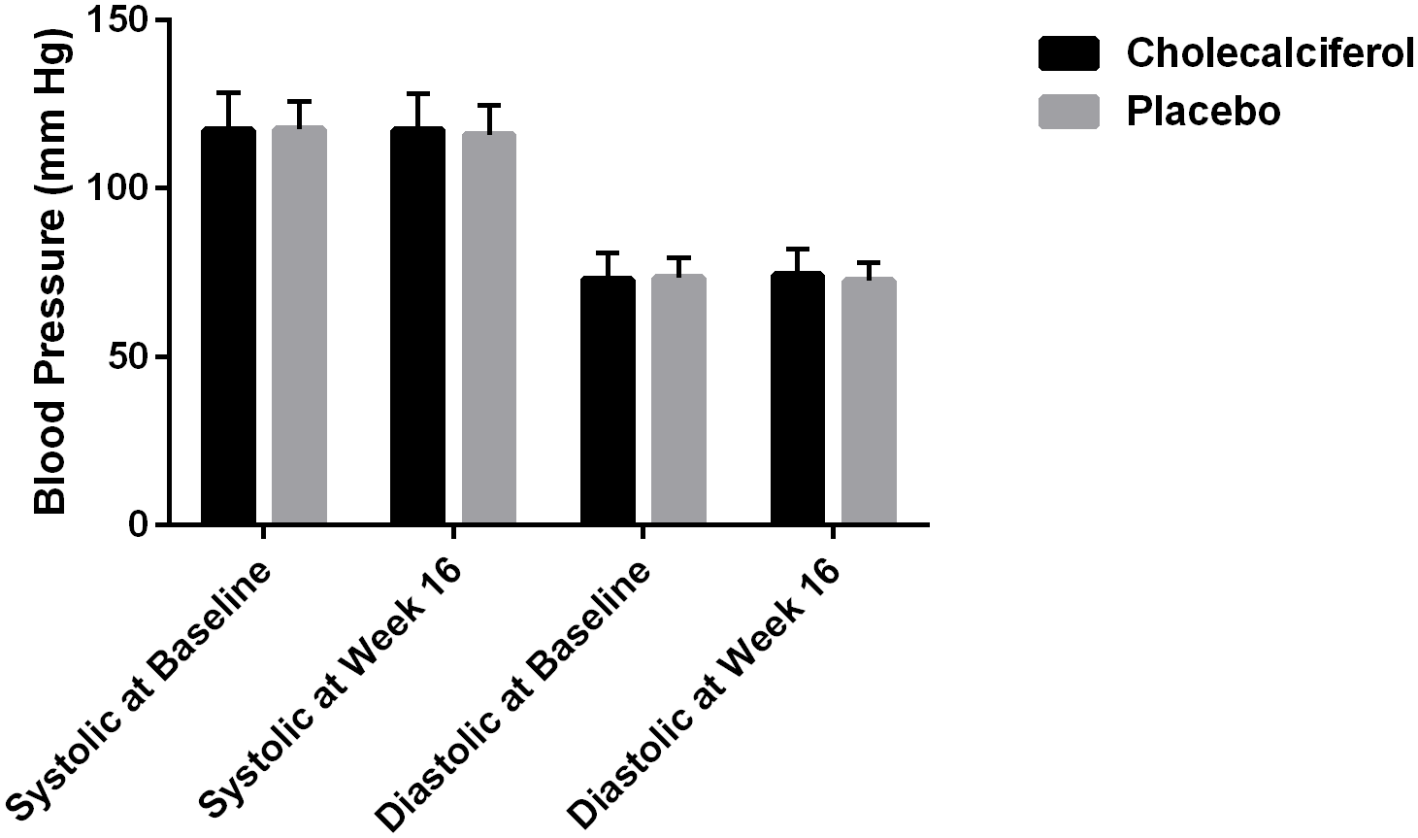
Treatment Effect on 24-hour Ambulatory Blood Pressure.

After 16 weeks of treatment there were no statistically significant differences in changes in PWV, AIx@HR, 24-hour ambulatory, peripheral or central SBP, DBP or PP between the two groups (Table 2 and Figs 3, 4 and 5). Within groups there was a statistically significant decrease in peripheral SBP of 5.2 mm Hg (95% CI: 2.2 to 8.2, p < 0.002) in the placebo group after 16 weeks of treatment (Table 2). There were no other significant changes in PWV, AIx@HR, 24-hour ambulatory, peripheral or central SBP, DBP or PP in either group after 16 weeks.

There were no significant correlations between changes in 25-OH vitamin D and PWV, AIx@HR, or 24-hour ambulatory SBP, DBP or PP (Table 3).

**Table 3:** Correlations between 25-OH Vitamin D and Vascular Stiffness and 24-hour Ambulatory Blood Pressure.

Table 3	*r*	p-value
*Δ*25-OH vitamin D vs. *Δ*PWV	0.156	0.337
*Δ*25-OH vitamin D vs. *Δ*AIx@HR	0.238	0.138
*Δ*25-OH vitamin D vs. *Δ*aSBP	0.094	0.562
*Δ*25-OH vitamin D vs. *Δ*aDBP	0.208	0.197
*Δ*25-OH vitamin D vs. *Δ*aPP	-0.112	0.490

aDBP = 24-hour Ambulatory Diastolic Blood Pressure, aPP = 24-hour Ambulatory Pulse Pressure, aSBP = 24-hour Ambulatory Systolic Blood Pressure, AIx@HR = Augmentation Index adjusted for Heart Rate, PWV = Pulse Wave Velocity.

## Discussion

We found no influence of 3000 IU oral cholecalciferol on arterial stiffness or blood pressure after 16 weeks of treatment. To the best of our knowledge, this is the first study to examine the effects of oral cholecalciferol on vascular stiffness in healthy adult individuals.

A previous trial using the same dose of oral cholecalciferol in subjects with hypertension found no difference in PWV or AIx after 20 weeks of treatment [22]. In that trial, 25-OH vitamin D deficiency was not an inclusion criterion, which could be the reason that no changes was found, however, another trial [23] examined the effects of a similar daily dose of oral cholecalciferol (2800 IU) on PWV in subjects with hypertension and 25-OH vitamin D < 75 nmol/L and also did not find an effect after 8 weeks of treatment.

Two other trials examined the effect of oral cholecalciferol treatment on PWV [24] and PWV and AIx [25] in subjects with type 2 diabetes mellitus using oral doses of 5000 IU daily and 2000 IU + 200 mg calcium daily, respectively. Neither study found any difference in vascular stiffness after 12 and 24 weeks of treatment, respectively. Both groups had 25-OH vitamin D < 35 nmol/L at baseline, despite vitamin D deficiency not being an inclusion criterion.

A fifth trial [26] examined the effect of oral cholecalciferol 2000 IU vs. 400 IU on PWV in black normotensive youths for 16 weeks in an open-label investigator-blinded randomized trial. There was a significant group by time interaction where PWV decreased in the cholecalciferol 2000 IU group by 0.08 m/sec, whereas the cholecalciferol 400 IU group had an increase in PWV by 0.33 m/sec.

For all of the above-mentioned trials PWV and AIx were secondary endpoints. However, all trials included more subjects than the 10 per treatment arm, which has previously been described as sufficient to detect a difference in PWV and AIx [18] and should therefore have sufficient statistical power to show any potential treatment effect. Since all of these trials including the present trial are of a relatively short duration, it is not possible to determine whether there are any long-term effects of cholecalciferol on arterial stiffness.

The effect of cholecalciferol on blood pressure has also been examined in several randomized clinical trials at various doses among various study populations [22–35]. The trials are relatively heterogeneous with regards to blood pressure, comorbidity, intervention (i.e. dose, formulation and frequency of cholecalciferol supplementation), follow-up, age, race and 25-OH vitamin D status. Overall, there seems to be either no or only modest blood pressure lowering effects of cholecalciferol supplementation, and a recent systematic review and meta-analysis of the blood pressure lowering effects of cholecalciferol supplementation [36] found no effect of treatment across various patient subgroups, and therefore advised against using cholecalciferol for treating hypertension. The results of our trial are in line with these conclusions.

The increased levels of both 25-OH vitamin D and 1,25-(OH)_2_ vitamin D in the cholecalciferol group suggests that the cholecalciferol was being adequately absorbed and converted to the biologically active compound and that the dose of cholecalciferol was adequate. However, there was also a slight increase in 25-OH vitamin D in the placebo group, which cannot readily be accounted for. Despite study participants being asked not to change their diet or ingest other mineral or vitamin supplements, we cannot exclude that dietary factors may have contributed to the increased levels of 25-OH vitamin D in either group. Also, travel activity to climates with more sun exposure or use of solarium were not recorded, and it can therefore not be excluded that factors other than the trial intervention caused the increases in 25-OH vitamin D. However, since there was a statistically significant difference in changes of 25-OH vitamin D and 1,25-(OH)_2_ vitamin D between the two groups any influence of this upon arterial stiffness should have been detected.

We detected no difference in arterial stiffness in the current study based on PWV and AIx@HR. However, we cannot exclude that other measures of assessing arterial stiffness might have been able to detect such differences, e.g. flow-mediated dilation, ultra sound imaging of the carotid intima/media thickness or coronary artery calcification score.

Subjects were healthy and well matched between the two groups making selection bias unlikely. Subjects were also normotensive and had normal PWV and AIx@HR. It could be speculated that an effect of cholecalciferol treatment would only affect subjects who had 25-OH vitamin D deficiency, since a subgroup analysis of a previous trial of cholecalciferol treatment for hypertensive subjects showed an effect in a subgroup of participants who were 25-OH vitamin D depleted at baseline [22]. In the current trial, however, baseline 25-OH vitamin D levels were 31 and 32 nmol/L for the cholecalciferol and placebo groups, respectively, making it unlikely that this was the reason for the lack of effect. What effect cholecalciferol might have on arterial stiffness and blood pressure in healthy subjects with very low levels of 25-OH vitamin D (e.g. ≤ 30 nmol/L) cannot be ascertained in the current trial. One might speculate that treatment with cholecalciferol would only be effective in subjects with various pathological states associated with arterial stiffness and hypertension (e.g. hypertension or increased RAAS activity), since basic science studies have shown that 1,25-(OH)_2_ vitamin D can suppress RAAS [16, 37]. However, a recent clinical trial of cholecalciferol treatment in hypertensive patients not on drugs affecting RAAS showed reductions in measures of RAAS, but no effects on blood pressure [30].

The strengths of this trial are its well-planned design and rigorous execution. Sample size calculations ensured that it was adequately powered to detect a difference in PWV and AIx@HR, although these were not the primary endpoints of the original trial. All data collection was done in a standardised manner and subjects were followed during the winter period in which the population in Denmark has the least sun exposure, thus limiting the effect of 1,25-(OH)_2_ vitamin D precursors other than the trial intervention.

However, this trial also has several limitations. First, although based on sample size calculations the trial should be large enough to detect a difference in PWV and AIx@HR, the sample size was still relatively small. Second, the follow-up time was relatively short. It is possible that a difference would be detected had the trial continued for a longer period of time. Third, although the two groups were similar at baseline, there were still slight differences between them, namely a slightly higher age, BMI and proportion of men in the placebo group. Since the trial investigated the effects of cholecalciferol treatment on surrogate end-points, we find it unlikely that the 3.5 years of difference in age would have a clinical effect. 25-OH and 1,25-(OH)_2_ vitamin D are both lipophilic and a higher proportion of fatty tissue (e.g. higher BMI) could theoretically lead to lower serum concentrations, which might limit the effects of the intervention. Women generally have a higher fat-to-muscle ratio then men, and therefore a higher proportion of women in the cholecalciferol group might have affected the effect of the intervention in the opposite direction toward lower serum concentrations of 25-OH and 1,25-(OH)_2_ vitamin D. Fourth, since the trial was performed during the winter period to limit the effects of sun exposure, the trial cannot answer the questions of whether there might be an effect of the intervention during other seasons or at different latitudes with greater sun exposure. Fifth, as previously mentioned, changes in diet, sun exposure or use of solarium were not recorded during the trial, and thus it cannot be excluded that these factors influenced the levels of 25-OH vitamin D and 1,25-(OH)_2_ vitamin D.

In conclusion, the results of this trial show that oral treatment with cholecalciferol 3000 IU daily for 16 weeks does not affect arterial stiffness or blood pressure in healthy normotensive human adults. Based on this we do not recommend using cholecalciferol for preventing arterial stiffness, however, long-term studies would need to be conducted to assess whether there are effects of cholecalciferol treatment on arterial stiffness and blood pressure in healthy adults treated for longer than 16 weeks.

## Supporting Information

S1 Text CONSORT checklist. (DOC)

S2 Text Trial Protocol (Original Danish Version). (DOC)

S3 Text Trial Protocol (English Version). (DOC)
