# Supplementary material for: The Effect of High Dose Cholecalciferol on Arterial Stiffness and Peripheral and Central Blood Pressure in Healthy Humans: A Randomized Controlled Trial
Source: PLoS One. 2016 Aug 10;11(8):e0160905. doi: 10.1371/journal.pone.0160905 (PMC4980002; doi:10.1371/journal.pone.0160905)
Supplement: S3 Text — (DOC) [file pone.0160905.s003.doc]

# Mineral metabolism, blood pressure and pulse wave velocity during vitamin D treatment

# (Mineralmetabolismen, blodtryk og pulsbølgemåling ved D vitamin behandling)

Ditte Hansen, MD, PhD Student

Niels Erik Frandsen, MD

Knud Rasmussen, MD DMSc

Lisbet Brandi, MD DMSc

Department of Medicine

Roskilde Sygehus

Køgevej 7-13

4000 Roskilde

Hans Christan Høck, MD PhD

Center for Clinical and Basic Research

**Background**

Several different patient groups have low levels of vitamin D, occasionally with disturbed mineral metabolism. The patients are advised to take vitamin D supplements. It is surprising, that even healthy individuals can have low levels of vitamin D, despite a normal diet and supplementation with vitamin D containing supplements [1,2].

There is not uniform consensus on the reference range for normal vitamin D levels [3]. Traditionally, plasma 25-hydroxy vitamin D levels > 50 nmol/L have been considered normal, since this is the level required for suppression of parathyroid hormone (PTH). Recently, there has been a tendency to increase this limit (e.g. 80 nmol/L) based on studies showing associations between high plasma 25-hydroxy vitamin D levels and increased bone density, prevention of bone fractures, reduced incidence of fall, increased muscle strength and lower incidence of colon cancer [4]. In order to reach such levels vitamin D intake of ≥ 1000 IU/day would be necessary [4,5]. Supplementation with vitamin D_3_ (cholecalciferol) results in a more stable serum concentration and is preferable to vitamin D_2_ (ergocalciferol) [6]. Previous studies of high dose vitamin D supplementation in subjects with osteoporosis have used combination treatment with vitamin D and calcium [7]. Vitamin D deficiency is associated with increased blood pressure and arterial stiffness [8,9] and some studies have shown an effect of vitamin D treatment on blood pressure [10,11]. Since vitamin D is categorised as dietary supplement it is therefore not submitted to the same strict approval process as new medication. In order to achieve levels of 25-hydroxy vitamin D levels > 80 nmol/L vitamin D supplementation is required, but prior to this it is necessary to investigate any adverse effects of such high doses, e.g. kidney stones due to increased urinary calcium and phosphate.

The purpose of this trial is to investigate the effects of high doses of cholecalciferol without calcium on mineral metabolism in healthy subjects, with particular focus on urinary calcium and phosphate. The trial will also investigate the effects on blood pressure and pulse wave velocity.

**Perspective**

Since large doses of vitamin D are being advocated to otherwise healthy people with low 25-hydroxy vitamin D levels it is necessary to investigate the effect on mineral metabolism. The kidneys' ability to handle calcium and phosphate is of particular interest if this treatment is to be used in patients with chronic kidney disease.

**Inclusion criteria**

1. Age > 18 years
2. Vitamin D insufficiency (P-25OHD < 50 nmol/l)
3. Written informed consent.

**Exclusion criteria**

1. Treatment with anti-hypertensive or anti-diabetic medication
2. Sarcoidosis
3. History of kidney stones
4. Treatment with 1α hydroxylating medication (e.g. Etalpha)
5. Plasma creatinine > 120 μmol/L
6. Ionised plasma calcium (mean of two measurements) > 1,50 mmol/L
7. Treatment with calcimimetics (e.g. cinacalcet)
8. Active malignant disease (active treatment or follow-up or diagnosed < 5 years ago)
9. Malabsorptive condition (including gastric bypass or bowel resection)
10. Active pancreatitis (plasma amylase > 3 times upper reference limit),
11. Previous hospital admission for alcohol-related disease
12. Actively illicit drug use
13. Pregnancy, breast feeding or unsafe contraception
14. Allergy to constituents of study drug

**Recruitment**

Subjects will be recruited through posters and information meetings at Roskilde University Hospital. Information will be available from week 36 of 2009 and inclusion will begin on September 1st 2009.

**Sample Size**

24-hour urinary calcium excretion in untreated subjects with osteoporosis is 5.0 ± 2.0 mmol [7]. Hypercalciuria is defined as 24-hour urinary calcium > 0.1 mmol/kg, i.e. approximately 7 mmol/24 hours for an adult person weighing 70 kg [12]. An increase in urinary calcium of 2 mmol is therefore a relevant minimal difference to detect. Since the variation between individuals is likely to be greater than the variation in a single individual, a standard deviation of 2.0 mmol/24 hours will be used to achieve a conservative sample size calculation. With a power of 90% and a 5% level of significance 20 subjects will be needed in each group to detect a difference in 24-hour urinary calcium of 2 mmol [13].

**Women of childbearing age**

Women of childbearing age included in the study must use safe anti-contraceptive therapy during the entire study period.

**Endpoints**

Primary

24-hour urinary calcium excretion (highest of two 24-hour measurements).

Secondary

24-hour urinary creatinine, protein, phosphate, sodium, potassium and uric acid; urine pH; blood pH; urine calcium/creatinine ratio; blood PTH, ionised calcium, phosphate, magnesium, 25-hydroxy vitamin D, 1,25-hydroxy vitamin D, triglycerides, fibroblast growth factor 23; clinic blood pressure; pulse wave velocity; 24-hour ambulatory blood pressure.

**Study medication**

Subjects will be randomized to 16 weeks of treatment with capsules of either cholecalciferol 3000 IE/day or matching placebo.

Cholecalciferol 3000 IU/day

n = 20

Placebo

n = 20

Randomisation

End of trial

16

0

week

baseline

**Study medication and process for randomisation and double-blinding**

Study medication and placebo will be delivered by D3 Pharmacy. Placebo tablets will be identical in appearance to the cholecalciferol tablets and contain the same constituents except cholecalciferol.

Each subject will receive 4 containers with 100 tablets in each, i.e. treatment for approximately 4-5 months.

Center for Clinical and Basic Research will pack and label the study medication based on a computer generated randomisation list. Container numbering will be numbered serially and will be administered to subjects in the order of their inclusion in the trial. Hans Christian Høck, department head at Center for Clinical and Basic Research, will keep the randomisation list. In the event of serious adverse events the randomisation list can be accessed by contacting Hans Christian Høck, and the reason for this will be recorded in the case report form.

**Side effects to study medication**

Supplementation with cholecalciferol at the dose used in this trial does not usually have any side effects. In a previous study of osteoporosis, patients treated with 18000 IE/day of cholecalciferol for five years did not have any increase in serum calcium. The risk of calcium stone precipitation in the kidneys (nephrocalcinosis) is deemed to be minimal and persons with reduced kidney function will be excluded from the trial.

**Compliance**

Compliance will be assessed by pill-count at the final follow-up visit.

**Medical treatment during the trial**

During the trial supplementation with other supplements is prohibited.

**Methods**

Laboratory analyses

A trial biobank will be established in order to perform experimental blood sample analyses in a single batch in order to minimise assay variation. Standard blood sample analyses will be performed immediately. Blood samples at weeks 0 and 16 will be drawn while subjects are in a fasting state with an extra 15 mL of blood placed in the biobank in case there will be a need for supplementary analyses. Each subject must have the same food intake during the two days of 24-hour urine collection and for three days prior to these. This will be recorded by a food intake diary.

Blood pressure measurements

Blood pressures will be measured using standard mercury sphygmomanometer according to the Danish Society of Hypertension Guidelines from 2006.

24-hour blood pressure

An ambulatory monitor, Spacelabs Medical, will measure the 24-hour blood pressure every 15 minutes at daytime (6 AM to 10 PM) and every 30 minutes at night (10 PM to 6 AM) over a period of 24 hours.

Pulse wave velocity

Measurement will be performed by applanation tonometry. A pencil-shaped high-fidelity micromanometer registers the intra-arterial pulse-wave by the general transfer function in a validated software program, SphygmoCor® (version 8.0, AtCor Medical, Sydney, Australia). The measurements will be calibrated by the brachial blood pressure and the augmentation index (AIx) will then be calculated from the central blood pressure curve. AIx is a measurement of the pulse wave amplification due to peripheral reflexion of the pulse wave. AIx is calculated as the difference between the first and second systolic peak as a percentage of the central pulse pressure (difference between central systolic and diastolic pressure). All AIx data will corrected for heart rate (AIx@HR). All measurements were done in duplicate and the mean of the two measurements was recorded. The Sphygmocor software provides a quality control of the recorded pressure waveforms.

Measurements will be performed after ten minutes rest in a calm environment and at a constant room temperature. The participants will not be allowed to speak or sleep during the examination, and will not be allowed to eat, drink or smoke three hours before or consume any alcoholic beverage ten hours before the examination, according to the present guidelines.

**Plan for study execution**

|  | screening | week 0 | | week 4 | | week 8 | | week 12 | | week 16 | |
| --- | --- | --- | --- | --- | --- | --- | --- | --- | --- | --- | --- |
| inclusion / exclusion criteria |  | X | |  | |  | |  | |  | |
| 24-hour urine collection |  | X | |  | |  | |  | | X | |
| Urine dipstick |  | X | |  | |  | |  | | X | |
| Blood samples: |  |  | |  | |  | |  | |  | |
| intact PTH |  | X^1^ | |  | |  | |  | | X^1^ | |
| ionised calcium | X | X | | X | | X | | X | | X | |
| phosphate |  | X | | X | | X | | X | | X | |
| magnesium |  | X | |  | |  | |  | | X | |
| 25-hydroxyvitamin D | X | X^1^ | |  | |  | |  | | X^1^ | |
| 1,25 dihydroxyvitamin D |  | X^1^ | |  | |  | |  | | X^1^ | |
| triglycerides |  | X | |  | |  | |  | | X | |
| cholesterol total/HDL/LDL |  | X | |  | |  | |  | | X | |
| FGF-23 |  | X^1^ | |  | |  | |  | | X^1^ | |
| creatinine | X |  | |  | |  | |  | | X | |
| amylase | X |  | |  | |  | |  | |  | |
| HCG (only fertile women) | X |  | |  | |  | |  | |  | |
| alkaline phosphatase |  | X | |  | |  | |  | | X | |
| Bone-specific alkaline phosphatase |  | X^1^ | |  | |  | |  | | X^1^ | |
| 24-hour ambulatory BP |  | X | |  | |  | |  | | X | |
| Clinic BP |  | X | |  | |  | |  | | X | |
| PWV |  | X | |  | |  | |  | | X | |
|  |  |  | |  | |  | |  | |  | |
| ^1^ Stored frozen in trial biobank | | |  | |  | |  | |  | |  |

**Statistics**

The primary endpoint will be assessed by an unpaired t-test and descriptive statistics will be performed for the total study population as well as for relevant regression analyses.

**Time Schedule**

Recruitment will begin in week 36 of 2009 and will continue until 40 subjects have been included. The trial is expected to conclude in May 2010.

**Location**

All procedures and biochemical analyses will be performed in the outpatient clinic at Roskilde Hospital and the Department of Biochemistry at Roskilde Hospital, respectively. All data will be recorded directly into a trial database.

**Rights and Publication**

All results, negative, positive and neutral, will be published in international peer-reviewed scientific journals.

**Ethics**

All information regarding individual study participants will be kept confidential.

Subjects will be informed of symptoms of hypercalcemia such as thirst and frequent urination. We consider this risk to be negligible due to the monthly follow-up of serum calcium. The study will provide significant information on the effects of vitamin D supplementation on mineral metabolism in the general healthy population.

**Financing**

Study medication will be provide free of charge by D3 Pharmacy, Denmark.

Analyses for 25-hydroxy vitamin D will be performed and paid for by Nordic Bioscience, Herlev Hovedgade 207, Herlev, Denmark.

Supplementary analyses will be paid for by the Division of Nephrology, Roskilde Hospital, and if possible through support from private or public funding.

Packaging and labelling will be performed free of charge by the Center for Clinical and Basic Research.

The study investigators are not affiliated with D3 Pharmacy, Denmark.

Subjects will not be paid for their participation in this trial.

References

1. Moller UK, Ramlau-Hansen CH, Rejnmark L, Heickendorff L, Henriksen TB, Mosekilde L: **Postpartum vitamin D insufficiency and secondary hyperparathyroidism in healthy Danish women.** *Eur J Clin Nutr* 2006, **60:** 1214-1221.

2. Mosekilde L: **Vitamin D and the elderly.** *Clin Endocrinol (Oxf)* 2005, **62:** 265-281.

3. Mosekilde L, Brot C, Hyldstrup L, Mortensen LS, Molgard C, Rasmussen SE *et al*.: **[The vitamin D status of the Danish population needs to be improved].** *Ugeskr Laeger* 2005, **167:** 895-897.

4. Bischoff-Ferrari HA, Giovannucci E, Willett WC, Dietrich T, wson-Hughes B: **Estimation of optimal serum concentrations of 25-hydroxyvitamin D for multiple health outcomes.** *Am J Clin Nutr* 2006, **84:** 18-28.

5. Hoeck HC, Li B, Qvist P: **Changes in 25-Hydroxyvitamin D3 to oral treatment with vitamin D3 in postmenopausal females with osteoporosis.** *Osteoporos Int* 2008.

6. Armas LA, Hollis BW, Heaney RP: **Vitamin D2 is much less effective than vitamin D3 in humans.** *J Clin Endocrinol Metab* 2004, **89:** 5387-5391.

7. Hasling C, Nielsen HE, Melsen F, Mosekilde L: **Safety of osteoporosis treatment with sodium fluoride, calcium phosphate and vitamin D.** *Miner Electrolyte Metab* 1987, **13:** 96-103.

8. Forman JP, Giovannucci E, Holmes MD, Bischoff-Ferrari HA, Tworoger SS, Willett WC *et al*.: **Plasma 25-hydroxyvitamin D levels and risk of incident hypertension.** *Hypertension* 2007, **49:** 1063-1069.

9. London GM, Guerin AP, Verbeke FH, Pannier B, Boutouyrie P, Marchais SJ *et al*.: **Mineral metabolism and arterial functions in end-stage renal disease: potential role of 25-hydroxyvitamin D deficiency.** *J Am Soc Nephrol* 2007, **18:** 613-620.

10. Lind L, Wengle B, Wide L, Ljunghall S: **Reduction of blood pressure during long-term treatment with active vitamin D (alphacalcidol) is dependent on plasma renin activity and calcium status. A double-blind, placebo-controlled study.** *Am J Hypertens* 1989, **2:** 20-25.

11. Pfeifer M, Begerow B, Minne HW, Nachtigall D, Hansen C: **Effects of a short-term vitamin D(3) and calcium supplementation on blood pressure and parathyroid hormone levels in elderly women.** *J Clin Endocrinol Metab* 2001, **86:** 1633-1637.

12. Escribano J, Balaguer A, Pagone F, Feliu A, Roque IF: **Pharmacological interventions for preventing complications in idiopathic hypercalciuria.** *Cochrane Database Syst Rev* 2009, CD004754.

13. Altman D.G.: *Practical Statistics for Medical Research*. 1991.
